# The Two Davidsons

**Published:** 1910-04

**Authors:** 


THE TWO DAVIDSONS.—Big D. (candidate for Governor) says: "I am certainly strongly and very heartily in favor of a law making suitable provisions for the treatment of [tuberculosis] such diseases and the appropriation of necessary money for the purpose. * * * Also, of the enactment of all other laws relating to and protecting the health of the people." Little D. (candidate for Lieutenant Governor) said: "Let the doctors dissect each other, damn them and their Ghost Bill."

---

NEEDLESS to say, I enjoy the "Red Back" immensely. I go through every number religiously and always find something peppery and pungent, and much that is worth while. No wonder that my Texas friends swear by Daniel—and that some people swear at him. I hope to have the pleasure of meeting you at the American Editors' meeting in St. Louis next June. You must try to be there. Come by all means.

Very truly yours,

W. C. ABBOTT.

---

IT IS a hard matter to get things fixed up to our liking, for the thing that the profession thought that they needed the worst of anything was a close organization; and now, alas, that has been turned into a means of oppression. Without the independent medical journals to keep track of their villainy, the medical profession would soon be a mere machine in the hands of a small coterie of medical politicians and wire-workers who would exploit it to their own aggrandizement.

Fraternally yours,

J. F. MCCARTY,
Comanche, Texas.

---

*My Dear Brother Daniel:*

God bless and prosper you.

J. E. GILCREEST,
Gainesville, Texas.

---

ROUGH FOR "SHE" BUT EASY FOR "HE."—Homer Clark Bennett, M. D., Lima, Ohio, contributes a poem to the *St. Joseph Medical Herald,* in which he says, "The road to reform is made easy for he who wanders away. * * * But the path is rough for she who sins," etc.

Now no one should be permitted to make it rough for "she." Professor Keene should write to the Octopus about it. This is only equaled by the little boy who said: "Her ain't a calling' of we; us don't belong to she."

